# Formation of Carbonized Polystyrene Sphere/hemisphere Shell Arrays by Ion Beam Irradiation and Subsequent Annealing or Chloroform Treatment

**DOI:** 10.1038/srep17529

**Published:** 2015-12-07

**Authors:** Xianyin Song, Zhigao Dai, Xiangheng Xiao, Wenqing Li, Xudong Zheng, Xunzhong Shang, Xiaolei Zhang, Guangxu Cai, Wei Wu, Fanli Meng, Changzhong Jiang

**Affiliations:** 1 Department of Physics and Key Laboratory of Artificial Micro- and Nano-structures of Ministry of Education, Hubei Nuclear Solid Physics Key Laboratory and Center for Ion Beam Application, Wuhan University, Wuhan 430072, P. R. China; 2 Faculty of Materials Science & Engineering, Hubei University, Wuhan 430062, P. R. China; 3 Laboratory of Functional Nanomaterials and Printed Electronics, School of Printing and Packaging, Wuhan University, Wuhan 430072, P. R. China; 4 Research Center for Biomimetic Functional Materials and Sensing Devices, Institute of Intelligent Machines, Chinese Academy of Sciences, Hefei 230031, P. R. China; 5 Su Zhou Institue of Wuhan University, Suzhou 215123, P. R. China

## Abstract

Heat-resistant two-dimensional (2D) sphere/hemisphere shell array is significant for the fabrication of novel nanostructures. Here large-area, well-ordered arrays of carbonized polystyrene (PS) hollow sphere/hemisphere with controlled size and morphology are prepared by combining the nanosphere self-assembly, kV Ag ion beam modification, and subsequent annealing or chloroform treatment. Potential mechanisms for the formation and evolution of the heat-resistant carbonized PS spherical shell with increasing ion fluence and energy are discussed. Combined with noble metal or semiconductor, these modified PS sphere arrays should open up new possibilities for high-performance nanoscale optical sensors or photoelectric devices.

The process of fabricating a large-area, well-ordered monolayer PS nanosphere array via diversified routes has been fully matured^1^,^2^,^3^,^4^,^5^. And the monolayer PS nanosphere array, whether as a mask or a substrate, combined with noble metal (particularly Ag and Au) or semiconductor-noble metal, acts as active platforms for surface enhanced Raman scattering (SERS) and there has been a large number of outstanding experimental results intended to describe the mechanisms of SERS and promote its application^6^,^7^,^8^,^9^,^10^,^11^. However, conventional monolayer PS nanosphere array has its inherent deficiencies such as poor tolerance of high temperatures^9^,^12^ and monotonical structures. At the same time, the as-prepared ordered array templates need to withstand high temperatures when they combine with other materials in many cases^13^,^14^,^15^.

Ion beam irradiation is one of the most powerful and well-known methods to modify the surface properties and topography of materials^16^,^17^,^18^. It has been demonstrated that, when organic polymer materials (e.g. PS, polymethylmethacrylate (PMMA)) are irradiated by a high-energy ion beam, some complex physico-chemical reactions will happen simultaneously, such as the effect of heat deposition, carbonization or cross-linking, surface sputtering effect, etc^19^,^20^,^21^,^22^. These reactions make organic polymer undergo drastic changes in morphology, composition and physicochemical properties. In particular, for high fluence (10^15^–10^17^ ions/cm^2^), a high degree of carbonization is observed with loss of the characteristic PS structure^21^.

In this work, as-prepared PS microsphere arrays are irradiated by kV Ag ion beam. We present a systematic investigation of the influence of the ion-irradiation parameters (ion energy, fluence) on the morphology and structure of PS microsphere array. After the subsequent annealing or chloroform process, we obtain large-area, well-ordered arrays of hemisphere shell or hollow sphere with small openings. With higher annealing temperatures, a nanobowl array is formed and the nanostructure can withstand heat treatment at 800 °C. These new structures overcome the deficiencies of conventional PS nanosphere array mentioned above and will surely facilitate further exploration of metal or semiconductor nanostructures for application in nanoscale photoelectric devices^11^,^23^,^24^,^25^.

## Experimentation

### Particle Hollowing Technique

**Figure 1:**
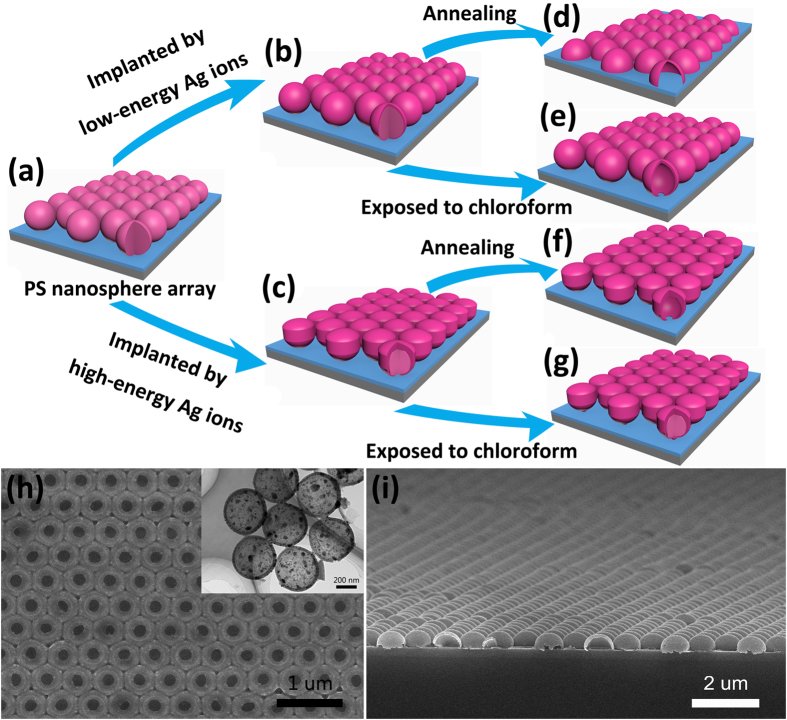
(**a–g**) Schematic illustration of the fabrication process of the ordered carbonized PS sphere/hemisphere shell arrays. (**h**) A top-view SEM image showing a well-ordered array of inverted hollow sphere with small openings 430 nm in initial diameter of PS sphere, and the inset is the corresponding TEM image. (**i**) A side-view SEM image showing a well-ordered hemisphere shell array 820 nm in initial diameter of PS sphere.

**Figure 2:**
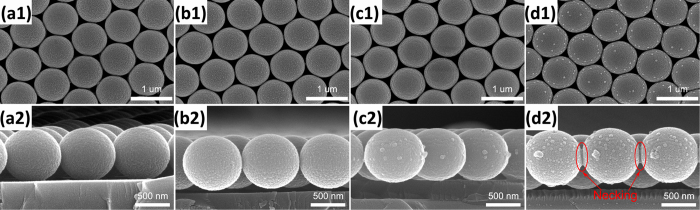
Top-view and higher magnification side-view SEM images of S1 (a1, a2),S2 (b1,b2), S3 (c1,c2), and S4 (d1,d2).

The fabrication process for the ordered array of carbonized PS hollow microsphere is illustrated in Fig. 1a–f. First, an ordered monolayer of PS microspheres was deposited on a silica substrate based on the Langmuir-Blodgett (LB) technique as described in literature^2^ (Fig. 1a). Second, the as-prepared PS microsphere array was irradiated with Ag ions vertically by a metal vapor vacuum arc (MEVVA) ion source implanter (Fig. 1b,c). During ion irradiation, the samples were kept rotating in a horizontal plane at a speed of 40 rpm and the sample stage was cooled by circulating cold water. The ion-irradiation parameters are listed in the Table 1. After ion irradiation, we obtained hollow microsphere array with different morphologies in two ways. One was to anneal the irradiated samples at 350 °C for 30 min in a conventional tube furnace in argon protective atmosphere (Figs 1d and 2f). The thermal treatment was also performed in air instead of argon atmosphere to investigate the influence of anneal atmosphere on the final PS microsphere structure. The other was that the irradiated samples were immersed in chloroform for a few minutes (Fig. 1e,g). To further research the thermostability of carbonized PS sphere shell, the annealing was carried out under higher temperatures (400, 500, 600, 800, and 1000 °C respectively) for 30 min. Figure 1h illustrates the SEM image of an ordered array of hollow microspheres (430 nm in initial diameter) with small openings and the inset is the corresponding TEM image. Figure 1i shows the side-view SEM image of a well-ordered hemisphere shell array for PS microspheres 820 nm in initial diameter. We can see the same large-area (25 × 20 μm), well-ordered, uniform-sized PS microsphere arrays and modified sphere shell arrays, as illustrated in Fig. S1 of the Supporting Information. We only show the research results of PS microspheres 820 nm in initial diameter below, even though we have obtained the similar results for 430 nm PS microspheres.

### Instrumentation

The as-prepared PS microsphere arrays were surface modified with Ag ions vertically by a MEVVA ion source implanter. The MEVVA ion source implanter was chosen for the implantation due to its high beam current capability, which greatly reduces the time and cost of sample preparation.

### Characterization

Scanning electron microscopy (SEM) and energy dispersive X-ray spectroscopy (EDS) were carried out using a FEI Sirion FEG scanning electron microscope at accelerating voltage of 20 kV. Raman scattering spectrum measurements were performed using a commercial Raman microscope (HR800, Horiba) and an Ar ion laser, emitting at 488 nm, was used as the excitation source. The molecular structure analysis of the irradiated and unirradiated PS was also characterized by using Fourier transform infrared spectroscope (FT-IR, Nicolet iS10, Thermo Fisher Scientific, USA). The microstructural characterizations were performed using a JEOL 2010 (HT) transmission electron microscope (TEM) operating at 200 kV.

## Results and Discussion

**Figure 3:**
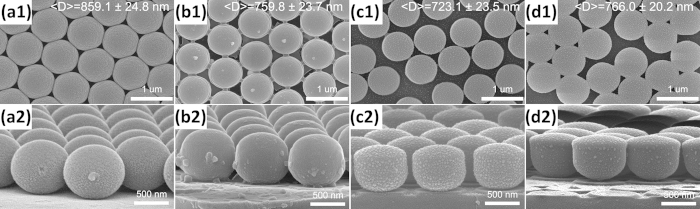
Top-view and higher magnification side-view SEM images of S5 (a1,a2), S6 (b1,b2), S7 (c1,c2), and S8 (d1,d2).

Figure 2a1–d1 are the top-view SEM images taken from the PS microsphere arrays irradiated by Ag ions at 15 kV with the fluences of 1 × 10^16^, 3 × 10^16^, 5 × 10^16^, and 7 × 10^16^ ions/cm^2^, respectively. Figure 2a2–d2 are the corresponding side-view SEM images. Figure 2 shows that the morphology and size of PS microspheres were almost unchanged with the increase of ion fluence. Only a coalescence phenomenon of neighboring PS microspheres occurred when the irradiated fluence reached 7 × 10^16^ ions/cm^2^. According to the existing studies about various methods for sintering colloidal particles^19^,^26^,^27^,^28^, the coalescence or neck formation results from the reduction of surface free energy under high temperatures. Therefore, we chose this irradiated fluence (7 × 10^16^ ions/cm^2^) in subsequent experiments and attempted to change the energy to investigate the influence of ion-irradiated energy on the morphology of PS spheres. With increasing irradiated energy, not only the morphology and size of PS sphere but also the structure of close-packed 2D hexagonal array have undergone dramatic changes, as shown in Fig. 3.

Figure 3a1–d1 show the top-view SEM images of irradiated samples with the irradiated energy of 10, 20, 40, and 60 kV, respectively. The corresponding side-view SEM images are given in Fig. 3a2–d2. It is observed that the top-view average size of PS particles increases from 820 ± 5 nm to 859.1 ± 24.8 nm, when the irradiated energy is 10 kV. Then the diameter of PS spheres continuously decreases to 723.1 ± 23.5 nm as the irradiated energy is increased to 40 kV. However, with irradiated energy further increasing to 60 kV, the diameter rises again slightly. It is also obvious that the hexagonal close-packed arrangement of PS spheres is broken and strong defect broadening by a collective motion of spheres is visible when the irradiated energy is higher than 40 kV (see Fig. 3c1,d1). Moreover, PS spheres are etched by kV Ag ions in a direction along the incident ions as shown in Fig. 3a2–d2. Especially, when the irradiated energy is higher than 40 kV, the original spherical PS particles become a nearly cylindrical shape after irradiation (see Fig. 3c2,d2). This etching phenomenon is totally different from the general etching of PS spheres by oxygen plasma^8^,^10^,^29^,^30^. Here it is worth noting that the silica substrates are also etched seriously by the kV Ag ions through the triangular voids (see Fig. 3a2–d2). This phenomenon directly leads to the final morphology and structure of spherical shells and we will discuss it in more detail later.

**Figure 4:**
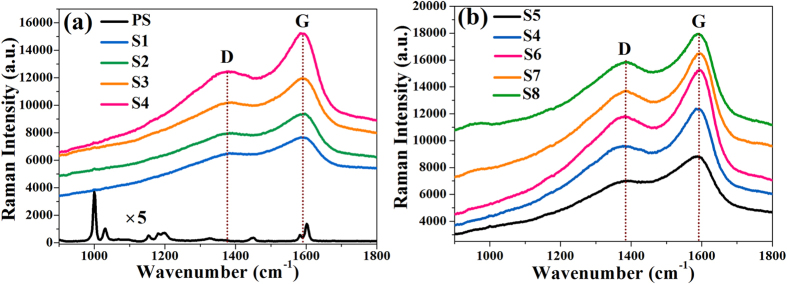
Raman spectra of unirradiated PS sphere array (PS) and Ag ions irradiated samples (S1–S8).

**Figure 5:**
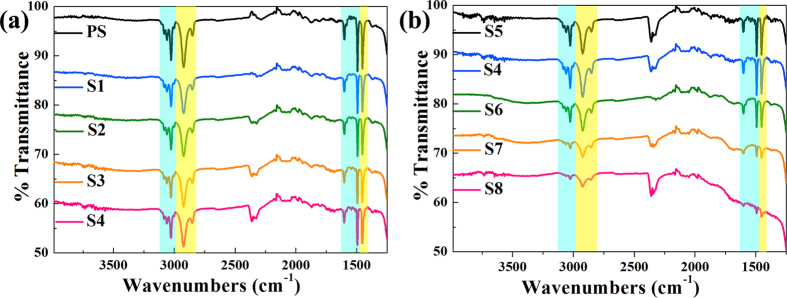
FT-IR spectra of unirradiated PS sphere array (PS) and Ag ions irradiated samples (S1–S8).

Here in order to find out the transmutation of chemical components occurring in the surface layer of the PS particle during ion irradiation, a Raman and FT-IR measurement were conducted, as shown in Figs 4, 5, respectively. The Raman spectrum of PS spheres array prior to ion irradiation shows lines at 1003, 1033, 1157, 1184, 1201, 1452, 1586, and 1605 cm^−1^, corresponding to vibrations of the polystyrene macromolecules (Fig. 4a). In contrast, the Raman spectra of irradiated samples only show intense, wide peaks at 1587 cm^−1^ and 1383 cm^−1^ as shown in Fig. 4. While the 1587 cm^−1^ peak corresponds to C=C stretching vibrations, its characteristic wavenumber is found in linear as well as cyclic π-conjugated systems. In contrast, the 1383 cm^−1^ peak originates from a hybridized vibrational mode associated with carbon edges, indicating the presence of some disorder in the C structure and its intensity relative to that of the 1587 cm^−1^ peak is indicative of the graphitization degree of carbon materials^20^. Following the model of Raman spectra for carbon structures^31^, the observed spectra of irradiated samples correspond to glassy carbon. This phenomenon, which is called carbonization or cross-linking, is consistent with previous studies^20^,^21^,^32^. The high fluorescence and background noise of irradiated samples may be related to a high concentration of defects generated during ion irradiation.

As shown in Fig. 5a, the FT-IR spectrum of unirradiated PS sphere array has characteristic lines at 3080, 3060, 3026, 1602 and 1493 cm^−1^, corresponding to aromatic ring vibrations and the aliphatic backbone of PS macromolecules appears on the spectrum as the absorption lines at 2923, 2852 and 1452 cm^−1^ (the absorption line around 2360 cm^−1^ can be observed on bare silica substrate). Meanwhile, the intensities of the absorption lines decrease slightly as the irradiated energy is 15 kV, but the peak strength remains almost unchanged with the increase of the irradiation fluence. These results are apparently different from Raman spectra shown in Fig. 4a. This may be explained by the phenomenon that the high background noise submerges the weak Raman signal of PS, which does not occur during FT-IR measurement. However, the peak intensity of FT-IR spectra decrease obviously with the increase of the irradiated energy (see Fig. 5b). This result can be interpreted as the size reduction of the PS spheres due to Ag ions etching and the increasing carbonization volume ratio of PS sphere.

**Figure 6:**
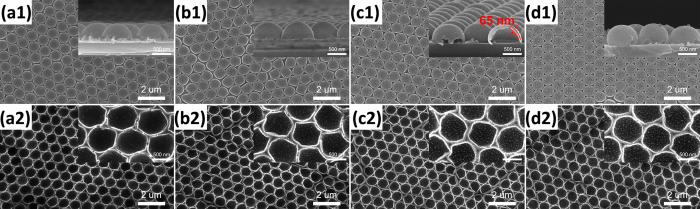
The top and bottom view SEM images of hemisphere shell arrays for modified samples of different irradiated fluence after thermal annealing at 350 °C for 30 min: S1 (a1,a2), S2 (b1,b2), S3 (c1,c2), and S4 (d1,d2). The insets in the images (**a1–d1**) show the corresponding higher magnification side-view SEM images. The insets in the images (**a2–d2**) are the corresponding higher magnification top-view SEM images.

**Figure 7:**
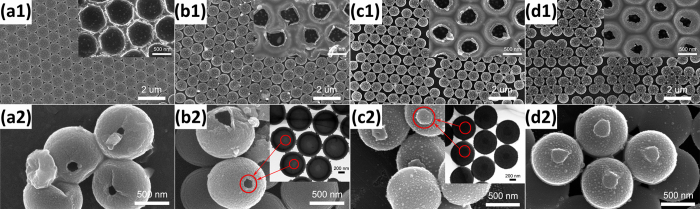
(**a1–d1**) The top-view SEM images of spherical shell arrays for modified samples of different irradiated energy after thermal annealing at 350 °C for 30 min: S5 (a1), S6 (b1), S7 (c1), and S8 (d1) and the corresponding higher magnification SEM images of the inverted spherical shell arrays. (**a2–d2**) The SEM images of PS spheres after exposed different energy Ag ions modified samples to chloroform for few minutes: S5 (**a2**), S6 (**b2**), S7 (**c2**), and S8 (**d2**). The insets in the images (**b2,c2**) are the corresponding TEM images.

Based on the spectroscopy analysis above, the thermal stability and the solubility in chloroform of modified PS were investigated in detail. According to the TGA (Thermogravimetric analysis) curve of polystyrene^12^, the annealing at 350 °C for 30 min under argon protective atmosphere was carried out first and the corresponding SEM images are shown in Figs 6 and 7a1–d1. Figure 6a1–d1 are the top-view SEM images of the annealed samples previously irradiated at 15 kV with the fluence of 1 × 10^16^, 3 × 10^16^, 5 × 10^16^, and 7 × 10^16^ ions/cm^2^, respectively. The insets show the higher magnification side-view SEM images. Figure 6a2–d2 are SEM images of inverted arrays shown in Fig. 6a1–d1, and the insets show the corresponding higher magnification SEM images. As given in Fig. 6, large-area, well-ordered hemisphere shell arrays are formed after annealing. The diameter and arrangement of hemisphere shells are almost the same as unheated ones, and the thickness of spherical shells is only a few tens of nanometers (see Fig. 6c1). Meanwhile, the unirradiated PS spheres undergo complete thermal decomposition and nothing is left under the same annealing condition (not shown here). According to the side-view SEM images of the hemisphere shells in Fig. 6, the vertical height of hemisphere shells has an obvious increasing trend with the increase of irradiated fluence, even though the viewing angle of cross-section images has some differences.

Figure 7a1–d1 show top-view SEM images of the annealed samples under the same annealing condition mentioned above which have been previously irradiated at 10, 20, 40 and 60 kV, respectively, to the same fluence of 7 × 10^16^ ions/cm^2^. The insets show the corresponding higher magnification SEM images of the inverted arrays. However, no hollow spheres are formed, if thermal treatment is performed in air as shown in Fig. S2 of the Supporting Information. Meanwhile, the Ag ion modified PS sphere arrays under the same irradiated parameters were exposed to chloroform for a few minutes. Then, we intentionally used tweezers to scratch the arrays gently to invert the spheres as shown in Fig. 7a2–d2. The insets of Fig. 7b2,7c2 show the corresponding TEM images.

Comparison between Figs 3a1–d1 and 7a1–d1 shows that the arrangement of irradiated PS spheres almost remains unchanged after annealing. However, with the increase of irradiated energy, the morphology of remaining shells after annealing changes from hemisphere shells to entirely spherical shells and the size of trepanning becomes smaller. Furthermore, it shows that trepanning occurs just where the PS sphere contacts the substrate. Meanwhile, the final morphology and structure of PS spheres of annealing and chloroform process are obviously different. For lower energy ion irradiation, hemisphere shells are formed after annealing, while entirely spherical shells with small openings are remained for chloroform treatment. And, when the irradiated energy is higher, the annealed samples generate spherical shells with small openings, while no hollow spheres are formed for with exposure to chloroform. (see the insets of Fig. 7b2,c2)

The following discussion will help us further understand the mechanisms for the formation of shells after annealing and chloroform treatment and the mechanisms for the influence of Ag ions fluence on the final morphology of PS spherical shells after annealing.

Combining SRIM (The Stopping and Range of Ions in Matter) calculations of the ions range^33^ (see Fig. S3 in Support Information) and the preceding spectral characterization (see Figs 4 and 5), we know that only surface layer of the irradiated PS undergoes carbonization, whereas the inner part keeps the polystyrene structure unaffected. The carbonized PS surface layer has a higher thermal decomposition temperature than the unchanged interior. Therefore, a hollow structure is formed for irradiated PS sphere arrays after annealing. According to the literature^21^, the untreated PS dissolved and could be washed away within a few seconds in chloroform, while the high-energy ions modified PS with high fluence could not be dissolved. So, we also obtain carbonized PS hollow spheres for irradiated PS sphere arrays after chloroform treatment as illustrated in Fig. 7a2,d2, even though the morphology and size of PS spheres do not change much with the increase of ion fluence at 15 kV Ag ions (see Fig. 2). However, it is seen that the higher irradiated fluence causes larger areas of PS sphere surface to be carbonized, finally resulting in larger areas of spherical shell to be left after annealing (see the insets of Fig. 6a1–d1). In summary, the irradiated fluence has some effects on the final morphology of spherical shells.

Meanwhile, description of the experimental results presented in Fig. 7 above reveals the influence of Ag ions energy and the subsequent process between annealing and chloroform treatment on the final morphology and structure of spherical shells. These results can be summarized as follows: (1) the hemisphere of PS particle close to the substrate (nether hemisphere) also undergoes cross-linking or carbonization during the ion irradiation; (2) the ion-irradiated degree of PS microsphere will directly affect its ability to resist thermal decomposition.

**Figure 8:**
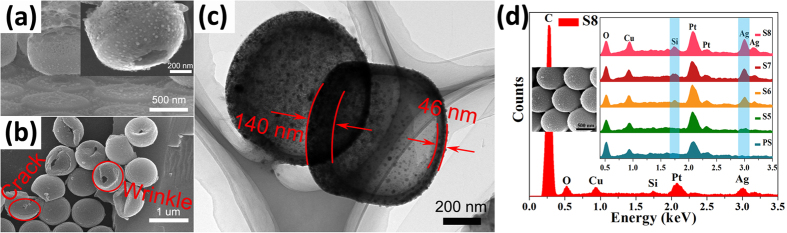
(**a**) The side-view SEM image of spherical shell array after thermal annealing of S7 and the corresponding higher magnification SEM image of a tilted spherical shell. (**b**) The SEM image of deformed spherical shells by exposing S4 to chloroform for few minutes. (**c**) The corresponding TEM image of (**a**). (**d**) EDX spectrum and the corresponding SEM image of inverted PS sphere array of S8 and the extracted EDX spectra of inverted PS sphere arrays of irradiated (S5, S6, S7, and S8) and unirradiated (PS).

**Figure 9:**
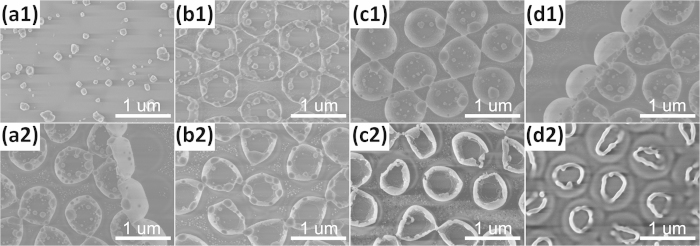
(**a1-d1**) The SEM images of modified samples of different irradiated energy after thermal annealing at 400 °C for 30 min: S5 (**a1**), S6 (**b1**), S7 (**c1**), and S8 (**d1**). (**a2–d2**) The SEM images of S8 after different temperatures annealing for 30 min: 500 °C (**a2**), 600 °C (**b2**), 800 °C (**c2**), and 1000 °C (**d2**).

To further clarify the mechanisms of these two kinds of phenomena mentioned above and research the upper limit temperature of Ag ion beam modified PS, more experimental results are given in Figs 8 and 9. Figure 8a shows a side-view SEM image of the annealed PS microsphere array which has been irradiated at 40 kV to the fluence of 7 × 10^16^ ions/cm^2^. The inset is a higher magnification SEM image of a tilted spherical shell and the corresponding TEM image is given in Fig. 8c. The PS sphere array irradiated at 15 kV with fluence of 7 × 10^16^ ions/cm^2^ after exposed to chloroform is also shown in Fig. 8b. Additionally, the energy-dispersive X-ray (EDX) analysis was used to probe the change of Si and Ag elemental composition of the inverted PS sphere arrays of irradiated and unirradiated, which were inverted from silica substrate to copper conductive tape. (see Fig. 8d and the inset is SEM image of the inverted PS sphere arrays of S8) The peak intensity of C element is too strong compared with other elements, and thus we wipe off C peak in the inset to highlight other peaks, particularly Si and Ag. (O and Cu elements mainly come from the copper conductive tape). In order to improve the conductivity of PS spheres arrays, a thin Pt layer was sprayed on samplers. Figure 9a1–d1 show SEM images of annealed samples at 400 °C for 30 min under argon atmosphere which have been previously irradiated at 10, 20, 40 and 60 kV, respectively, to the same fluence of 7 × 10^16^ ions/cm^2^. Nanobowl array is formed for 60 kV Ag ions beam irradiated PS particles after annealing. Therefore, the annealing of higher temperatures (at 500, 600, 800 and 1000 °C respectively, for 30 min) was conducted for this parameters PS sphere array. The corresponding SEM images are given in Fig. 9a2–d2.

Combining the SRIM calculations of the ions range (Fig. S3 in Support Information) and the final hollow structure of irradiated PS sphere after annealing, we can be sure that the cross-linking or carbonization of nether hemisphere cannot be caused by penetrating ions. As shown in Figs 3a2–d2 and 8a, the silica substrates have been etched seriously by kV Ag ions through the triangular void. Part of the sputtering atoms also possess very high energy compared with the binding energy of atoms in PS and the energy radiates to the surrounding space^34^ finally leading to the cross-linking or carbonization of nether hemisphere. The sputtered atoms come from the substrate material and the previously implanted Ag ions. Therefore, we can observe the appearance of Ag nanoparticles on the nether hemisphere surface for irradiated sample after annealing (see the inset of Fig. 8a). This explanation is apparently consistent with the experimental observations that the appearance of Si element for irradiated samples during the EDX analysis. To assist understanding, a schematic diagram is shown in Fig. S4 of the Supporting Information.

For the second phenomenon about the carbonization or cross-linking degree of PS affecting its ability to resist thermal decomposition, we give some more evidence and explanations below. The energy of sputtering atoms is much lower than that of irradiated ions, and the energy distribution of sputtering atoms moves towards the high-energy region with the increase of irradiated ion energy^34^,^35^. This has also been supported by the TEM image shown in Fig. 8c. Figure 8c shows that the spherical shell thickness is not uniform and the thickness of the hemisphere shell (upper hemisphere shell) carbonized by directly irradiated Ag ions is much larger than that of the hemisphere shell close to the substrate (lower hemisphere shell) carbonized by sputtering atoms. The deformation of the spherical shells also differs between both hemisphere shells (see Fig. 8b). The distortion of the lower hemisphere shell shows good flexibility while the upper hemisphere shell directly cracks under the effect of outside force. The results of higher annealing temperatures (at 400 °C for 30 min) show that only some Ag nanoparticles are left for PS modified with the lower energy beam, while nanobowl arrays are formed for PS (see Fig. 9a1–d1) irradiated with the higher energy beam. These experimental results are also exactly consistent with the second phenomenon mentioned above. But the upper hemisphere shells show higher thermal stability for PS sphere (see Fig. 6) modified with the lower energy beam; however, the lower hemisphere shells have better heat resistance as irradiated energy is higher (see Fig. 9). Therefore, it is not a simple proportional relationship between the ability to resist thermal decomposition and the ion-irradiated degree of modified PS surface layers. Figure 9a2–d2 show that the nanobowl arrays can withstand heat treatment temperature as high as 800 °C without structural damage, even though Ag nanoparticles are removed due to thermal evaporation. This result indicates that the existence of Ag nanoparticles does not affect the thermostability of modified nanobowl arrays.

## Conclusions

In conclusion, we have successfully prepared large-area, well-ordered heat-resistant carbonized PS sphere/hemisphere shell arrays in a controlled way. By adjusting the irradiated Ag ion energy and fluence, effective control of PS sphere morphology and size has been achieved. With subsequent annealing or chloroform treatment, sphere/hemisphere shell arrays have been formed. The possible mechanisms have been discussed in detail. The carbonization effect and surface sputtering effect are considered the main reasons for forming the final spherical shell arrays. By kV Ag ion beam modification, our work diversifies the structure and morphology of PS sphere array and greatly improves its thermostability, paving the way for the application of PS nanosphere array in nanoscale photoelectric devices.

## Additional Information

**How to cite this article**: Song, X. *et al.* Formation of Carbonized Polystyrene Sphere/hemisphere Shell Arrays by Ion Beam Irradiation and Subsequent Annealing or Chloroform Treatment. *Sci. Rep.* **5**, 17529; doi: 10.1038/srep17529 (2015).

## Supplementary Material

Supplementary Information

## Figures and Tables

**Table 1:** Silver irradiation parameters for all samples.

Sample	Energy of ion implantation (kV)	Dose of ion implantation (×10^16^ ions/cm^2^)
S1	15	1
S2	15	3
S3	15	5
S4	15	7
S5	10	7
S6	20	7
S7	40	7
S8	60	7
