# Supplementary material for: Formation of Carbonized Polystyrene Sphere/hemisphere Shell Arrays by Ion Beam Irradiation and Subsequent Annealing or Chloroform Treatment
Source: Sci Rep. 2015 Dec 7;5:17529. doi: 10.1038/srep17529 (PMC4670998; doi:10.1038/srep17529)
Supplement: Supplementary Information [file srep17529-s1.doc]

**Formation of Carbonized Polystyrene Sphere/hemisphere Shell Arrays by Ion Beam Irradiation and Subsequent Annealing or Chloroform Treatment**

Xianyin Song^1^, Zhigao Dai^1,3^, Xiangheng Xiao^1,5^*, Wenqing Li^1^, Xudong Zheng^1^, Xunzhong Shang^2^, Xiaolei Zhang^1^, Guangxu Cai^1^, Wei Wu^3^, Fanli Meng^4^,*, Changzhong Jiang^1^

1 Department of Physics and Key Laboratory of Artificial Micro- and Nano-structures of Ministry of Education, Hubei Nuclear Solid Physics Key Laboratory and Center for Ion Beam Application, Wuhan University, Wuhan 430072, P. R. China

2 Faculty of Materials Science & Engineering, Hubei University, Wuhan 430062, P. R. China

3 Laboratory of Functional Nanomaterials and Printed Electronics, School of Printing and Packaging, Wuhan University, Wuhan 430072, P. R. China

4 Research Center for Biomimetic Functional Materials and Sensing Devices, Institute of Intelligent Machines, Chinese Academy of Sciences, Hefei 230031, P. R. China

5 Su Zhou Institue of Wuhan University, Suzhou 215123, P. R. China

^*^ Authors to whom correspondence should be addressed. Electronic addresses: [xxh@whu.edu.cn](mailto:xxh@whu.edu.cn), flmeng@iim.ac.cn

**Supplementary Material Includes:**

**Figure S1.** (a) The digital picture of monolayer colloidal crystals. (b) SEM image of as-prepared large-area (25×20 *μ*m), well-ordered, uniform-sized PS microsphere array. (c) SEM image of carbonized PS hemisphere shell array after Ag ion beam modification and following annealing. The inset is the corresponding higher magnification SEM images.

**Figure S2.** (a-d) The top-view SEM images of ion beam modified samples of different irradiated energy after thermal annealing at 350 °C for 30 min in air: S5 (a), S6 (b), S7 (c), and S8 (d).

**Figure S3.** Calculated depth profile and projected range of the implanted Ag ion in PS by SRIM. The implantation has been carried out at energy of 60 keV to a dose of 7 × 10^16^ ions/cm^2^ (a) and at the energy of 10, 15, 20, 40, and 60 keV, respectively (b).

**Figure S4.** The schematic diagram is for the evolution of polystyrene (PS) microsphere during irradiation.


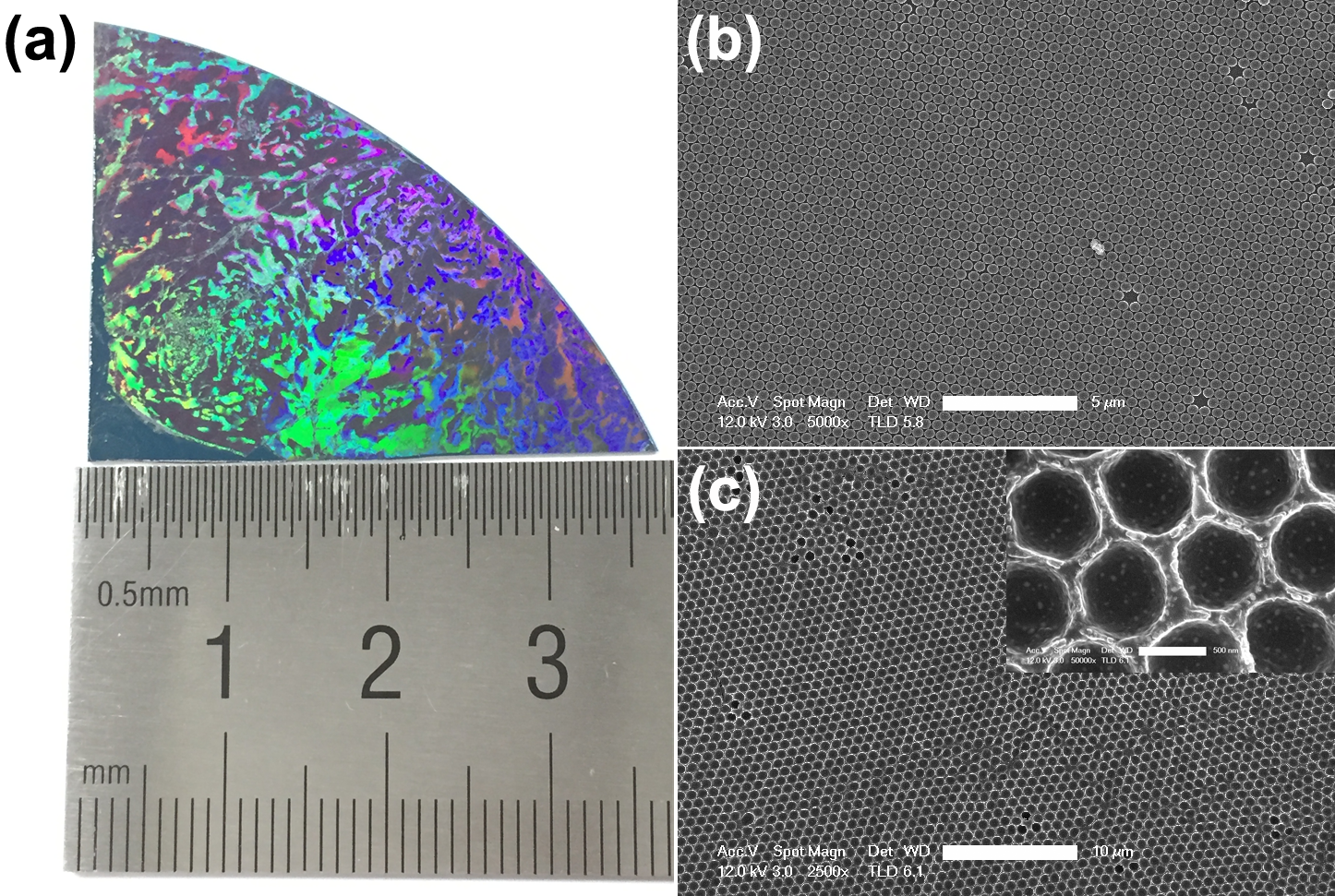


**Figure S1.** (a) The digital picture of monolayer colloidal crystals. (b) SEM image of as-prepared large-area (25×20 *μ*m), well-ordered, uniform-sized PS microsphere array. (c) SEM image of carbonized PS hemisphere shell array after Ag ion beam modification and following annealing. The inset is the corresponding higher magnification SEM images.


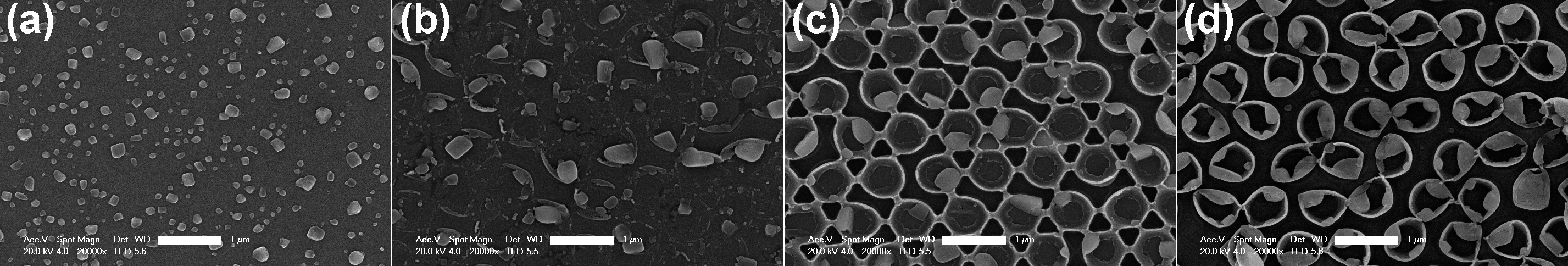


**Figure S2.** (a-d) The top-view SEM images of ion beam modified samples of different irradiated energy after thermal annealing at 350 °C for 30 min in air: S5 (a), S6 (b), S7 (c), and S8 (d).

**
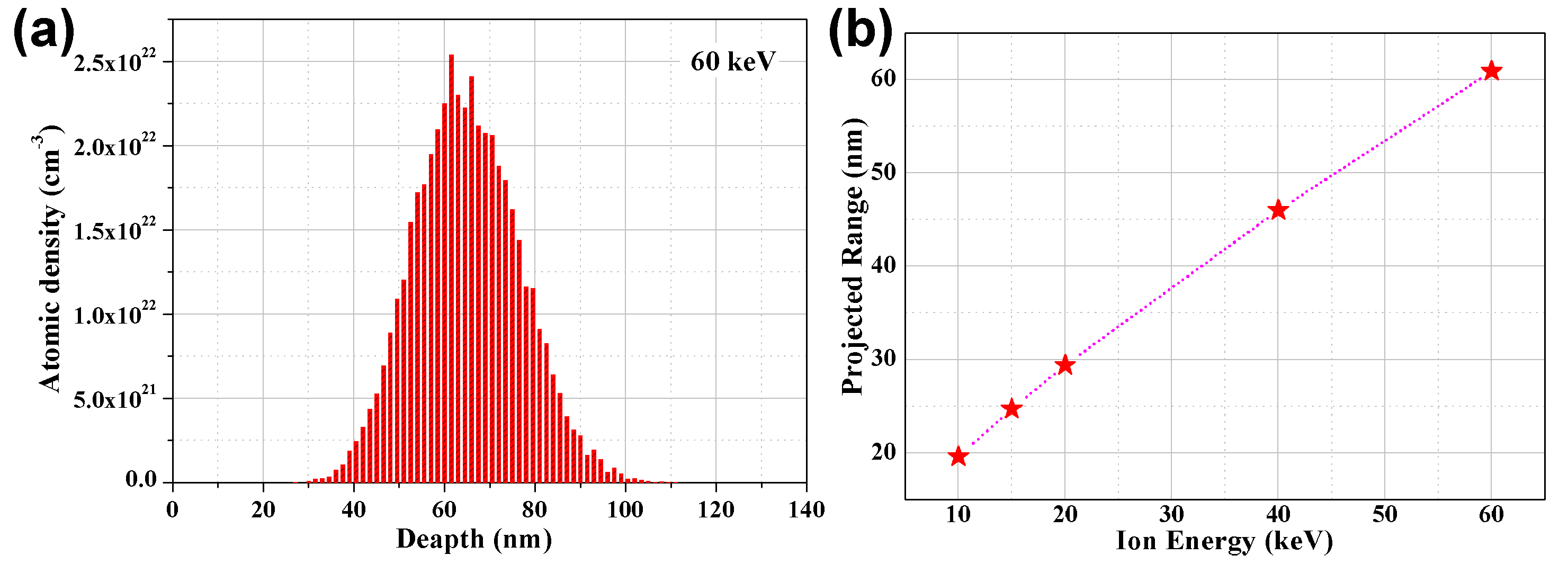
**

**Figure S3.** Calculated depth profile and projected range of the implanted Ag ion in PS by SRIM. The implantation has been carried out at energy of 60 keV to a dose of 7 × 10^16^ ions/cm^2^ (a) and at the energy of 10, 15, 20, 40, and 60 keV, respectively (b).

**
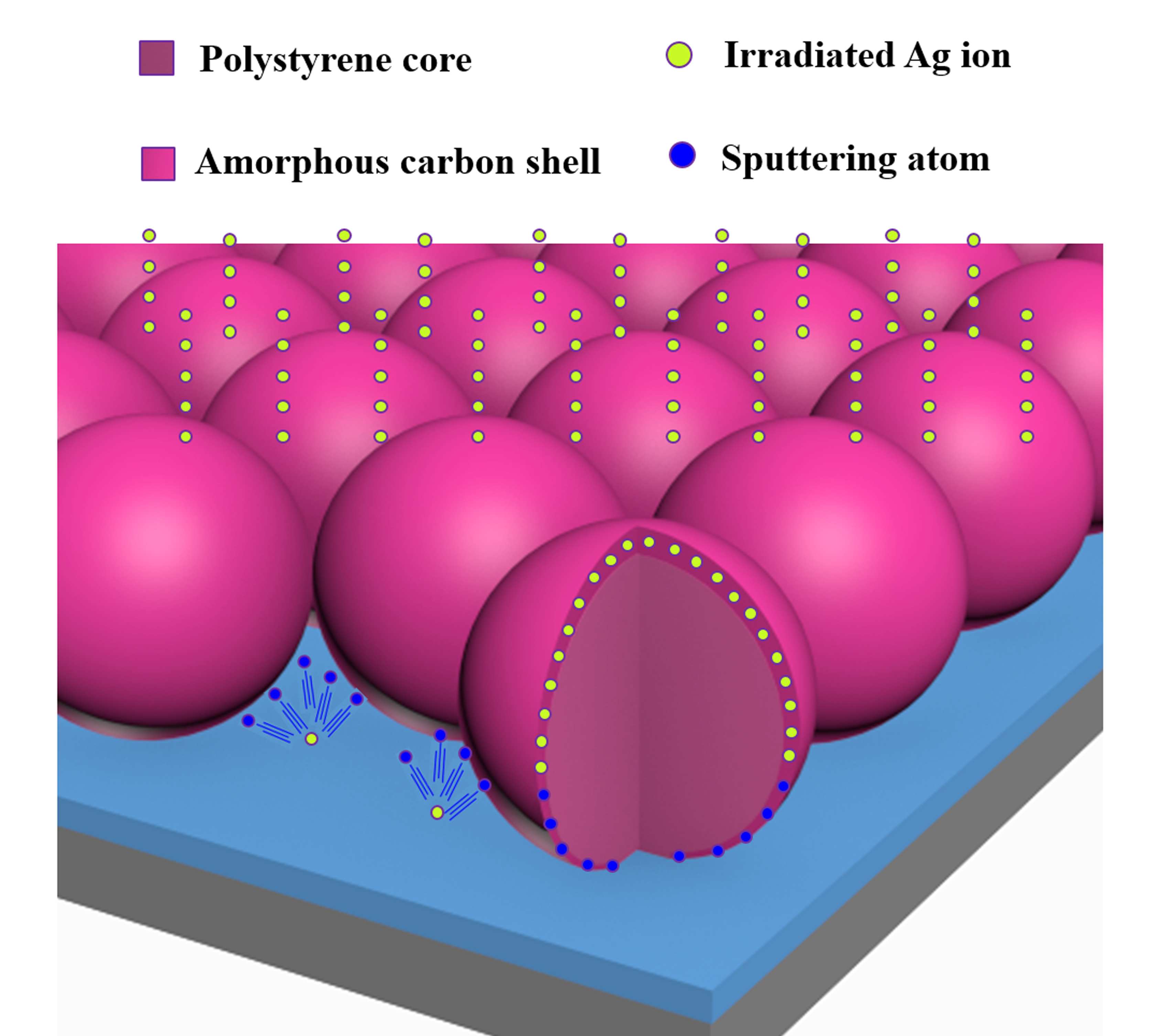
**

**Figure S4.** The schematic diagram is for the evolution of polystyrene (PS) microsphere during irradiation.
